# Diet and the Prolongation of Life

**Published:** 1913-07

**Authors:** John C. Warbrick

**Affiliations:** Chicago, Illinois


For Texas Medical Journal.

### Diet and the Prolongation of Life.

BY JOHN C. WARBRICK, M. D., CHICAGO, ILLINOIS.

From the earliest times up to the present, fads of some kind have always existed, and so have faddists, and they are likely to continue to exist for many ages to come. As one generation succeeds unto another, so the propagation of one fad may continue after another has died out, not only by individuals and groups of them, but also by societies which have something in common that appeals to their liking or personality. Fads, of course, are not limited to any one field in particular that is adopted by all, but embrace a large variety of things, each one drawing adherents to its cause as fancy appeals to them.

With regard to diet, possibly nothing has been more ridden for years nor discussed so much as to what is the best food to take, while, at the present time, nothing seems to be more talked about nor more ideas suggested in connection therewith, than that which concerns one's health and what is the best thing to prolong life to a good old age. This seems to be only quite natural, however, for every individual is more or less interested in what concerns his bodily welfare and any measures suggested to that end, will, of course, attract attention.

As to the proper dietary to follow, and the prolonging of life to a good old age, opinions are varied and numerous—for what is good for one man may be disagreeable to another, according to the desires, tastes, temperament and habits of individuals which differ a good deal, so that to begin to lay down a solid foundation of facts suitable for *every* individual, is almost impossible.

Since the beginning of time mankind has managed to exist on the ordinary articles of diet and to thrive thereon. Wonders have been accomplished by the ingenuity of man and science has been transformed by masters who lived on the ordinary diet. If it was good enough for our forefathers, it is surely sufficient for the present—while future generations will reap the benefit of it, for it has been proved in a multitude of instances that a great age can be reached and good health secured, without adhering strictly to any special or dietetic regimen. Numerous instances can be pointed out where remarkable age has been attained by the ordinary mode of living and without any special fads or precautions being taken to safeguard the health during life. Many of these mental and dietetic fads and fancies play no part in prolonging human life but very often do a lot of injury, as has been proved in many instances and which are noted later on in this article, but serve merely as distractions for the temperament the time being for those individuals who may require them.

Among the various methods stated as to what is the best diet to follow and live to a great age, are the following:

1. One advises the public to follow vegetarianism and live to be 200 years old by taking only two meals a day, dinner and lunch. No flesh food in the diet whatever is allowed but articles such as rice, whole wheat bread, vegetables, nuts and fruits. No coffee, tea, cocoa, liquors or rich foods, must be taken. No salt, because it makes the bones stiff, as is stated. Nothing to drink at meals but only between meals. Deep breathing and moderate exercise complete the regimen.

2. A number advise against the use of meat as being both poisonous and injurious. Among these are such well known persons as Mrs. Ella Wheeler Wilcox, Dr. Kellogg, of Battle Creek, Michigan, Dr. Haig, of England, and George Bernard Shaw, of England, Mr. Horace Fletcher, who holds the record for two performances, and Bernarr McFadden, London.

3. Some person suggests cheese, which is as nourishing as butcher's meat and less expensive.

4. Another one says eat onions in order to live long. The woman who advises this, lived to be 115 years old, and began eating them when she was young, ascribing her longevity to them.

5. Every ailment being due to the habit of sitting, it seems the advice given is to abolish it entirely and have people stand. If in need of rest people should recline for a time on their backs; and from doing this they are supposed to be free from illness and live to old age.

6. To live sanely and moderately. To be temperate in habits. To keep sweet and live in the present, is the advice of a man who has passed ninety.

7. A French surgeon claims that a substance called "Mycolysine" will prolong life and is the elixir looked for to perpetuate existence.

8. Then we have a statement that by doing certain things we should live forever.

9. From Johannesberg, South Africa, we learn that Mr. Williams, a lawyer, tested Upton Sinclair's fasting cure for dyspepsia and died.

10. Be a vegetarian.

11. Do not smoke.

12. Take out-door exercise.

There are then, a number of varied statements made as to what is the best plan to follow to maintain health and to prolong life to a well advanced age. Some, who have gotten a good deal beyond the allotted span of life,—three score years and ten,—give advice to others as to how to reach a ripe old age, so that all kinds of ways are suggested. Because certain individuals have enjoyed good health in one way, have reached a good old age with a certain constitution, it is then taken for granted that every one else could be equally as healthy by doing the same thing; and it is heralded abroad that the "Golden Age" has been reached. Some individuals, no matter what they did, nor how hard they tried, could never reach a moderate old age because their constitutional make-up forbids it. Their allotted years of life have been fixed at much less by physical conditions. There are others who are destined by the ordinary means of living to reach a high index figure for physical conditions make it that way, unless something unforeseen overtakes them at any time; in fact, it may be said that every person has it stamped upon his physique the manner and mode of death and every physique bears it. There are a great many erroneous statements made by various persons as to what is the proper means to maintain health and prolong life to a good old age; each one having a different idea according, it would seem, to his mental and physical make-up.

When a person is young and active, tissue change is more rapid, food is digested quicker, there is freedom from worry, and when middle life is reached less activity is indulged in, as a rule, and the habits are more sedentary; while tissue change is not so great as they are more firm and not so elastic. From youth to old age too much food is taken for the requirements of the body; this has been proved from the examination of a great many urinary specimens very carefully, when the chlorides, as a rule, have been in most instances far beyond the maximum normal daily amount. However, to measure out for each meal the exact amount of food required for the body would only make life a bore, and eating a dread, but, as it is, humanity manages to get along pretty well, judging from the fact of how numerous are the people who reach a good old age and who have simply followed out the ordinary rules of living.

It is not so much the *kind* of food that one takes from time to time that is harmful, although that may be injurious enough, but it is the *quantity*, according to requirements, and as the continued eating of heavy meals, bolting the food, insufficient chewing, the wrong kind of foods, etc. When the habit of eating heavy meals has been contracted it is not easy to stop it, besides, certain people have not the resisting powers of the temperament to do so, the same as others. Heavy meals are not necessary; a restricted diet is better than a heavy one, for all kinds of work can be done better on lighter meals than on heavier ones. If more food is taken than the system demands, it will decompose and poison the body unless certain conditions are followed out in taking care of the body so it can be got rid of.

No one can do himself much harm by under-eating, but can do himself a great deal of harm by over-eating continually. Consider upon what a very little the Japanese live and maintain a splendid physique, capable of hardships and endurance that it is difficult to surpass, especially among the soldiers. The Japanese, of course, according to their customs, physique, training, and nationality have their own way of living that is different to that of other nations, and could not be wholly adopted by any of them, yet, people who live in Japan could be counted by the hundreds who have reached a good old age by plain living and eating.

If we go to Scotland we find the Scotch, especially the regular troops, splendid specimens of physical manhood, and the same of the peasants. We find the Scotch very hardy and a long-lived race, having their own way of living on porridge, oatmeal, bread, cheese, etc. Thrift, along with this, as the Scotchman has strong characteristics that mark him; then the air, from the healthy hills and dales, no doubt also helps some.

In Ireland we find people reach just as old an age as in any part of the world, and we find they live a good deal on potatoes, bread, tea, etc., and live a simple life. There are fine specimens of manhood found among the Irish. We may go from one country to another: Russia, Germany, Sweden, Norway, Switzerland, France, Persia, Turkey, Arabia, etc., where the habits and diets of the people are different, and find splendid specimens of manhood among them all; and in each country—in fact all over the earth—we may find people who have lived and are living to a great age, not by Christian Science or any special methods or fads —but by the ordinary methods of living in vogue in their own country for many centuries.

In order to prove that no special diet or fad are necessary, to prolong life to a good old age, it is only necessary to report the cases of a number of men and women who have reached the century mark—while others have passed three score years and ten, by simply the ordinary means of living. A great deal has been written about how to live to a good old age. There have been sent forth numerous formulas given for years as to the real elixir of life, but of no use. Life flows on just the same while just as many people die and just as many reach an old age as ever, for the rules that govern one constitution in reaching old age do not govern that of another; while the vast majority of people pay little or no attention to the fact of prolonging life to a good age by some special means. It may be it was never intended for many to reach the century mark, which very few do—hardly two in a million. It is said that 68 people die every minute, or about

35,000,000 per year, so no matter what method one follows or what is taken, it is bound to go on just the same. It would be almost useless to review the different methods the thousands who have reached a ripe old age have followed, but there is a similarity in nearly all of them; in most cases and in many ways, for they have all breathed the same air, they have all slept, whether lightly or soundly—at night; they have all taken much the same kind of food, whether meat or vegetables. They have all taken water, more or less; they have all had exercise both of the mind and body; one has smoked all of his life, and says the use of tobacco has prolonged it, while another states the reverse, and believes if one wants to live to a great age he must not smoke. In the word *"worry"* we possibly have the key-note to the reason why many fail to reach old age, for peace of mind is, no doubt, the essential of all things for the body welfare, in prolonging life, as it is for almost every undertaking. It rides the body over all obstacles safely, while worry tends to hinder it and causes ill health. When the mind is master of the body much can be done, but where the body is master of the mind, a great deal cannot be done.

From the report of the following 19 cases it will be seen that a remarkable age has been reached in each instance by the ordinary means of living, and these are only a few cases out of many thousands who have attained a remarkable age. We venture to say that none of those whose names are previously mentioned in this article, who strongly denounce certain articles of diet as being injurious and poisonous, will ever reach anywhere near the lowest age figure quoted in the reports of these cases, neither will any of them who ardently propagate fads get any where near any of the figures mentioned. From glancing over these cases it will be seen that hardly any other evidence is necessary to prove that Christian Science would be a failure in trying to prolong life to any such ages as those noted.

1. Mrs. J. D., of Marshall county, Illinois; mother of ten children, is over 106 years old. She says: "Don't worry."

2. Dr. P., 100 years old and in good health; 11 children; no pain and never worries; chops and saws wood daily; walks and takes out-door exercise daily, which he says has caused him to live to a good old age.

3. Mrs. R., 106 years old; has no need of glasses and hears as well as if young; 8 children; mental faculties clear; never was sick.

4. W. J. F., 101 years old; feels just as good as at 19; only once had any severe illness; stubborn determination to reach the century mark.

5. Mrs. W., 101 years old; the eleventh child in a family of 23; 18 grew to manhood and womanhood; healthful, normal life; most of it spent in the country, to which she attributes her advanced age. Worries and strains in early days, then peace and content later.

6. E. F. runs a farm at 100 years old; gets out of bed at 5 o'clock every morning and cares for horses and cattle before breakfast; says he would not have reached 100 had he not kept from tobacco and alcohol. Is active and healthy.

7. J. M., 107 years old; always violated rules advanced to those who desire long life; fond of pipe and smokes yet; spent most of life out doors and walked a good deal.

8. E. P., 102 years old; used tobacco all life; mother lived to 105 years; father chopped wood after 102.

9. Rev. J. C., 101 years old; never was ill; eats nothing but dry bread two days old; fresh fruit and black tea; mind agile and keen. Says, "Do good and aim high and the rest will take care of itself."

10. J. H., 93 years old; retired carpet weaver; full possession of all his mental faculties; no special illness of any kind; always went to bed in good season and rose early. Simple and regular in his habits. For years worked in a small room with no ventilation; took pork and other kinds of meat; always contented and never worried; always slept well; never had a headache; never wore glasses nor smoked.

11. Mrs. W. died at age of 96; always had good health; lived in the country all of her life and never had any fads; lived plainly and had a calm mind. Never worried about her food. Use of her mental faculties to the end.

12. J. A., farmer; lived to be 91; always worked hard and had no fads. Disposition bright and cheerful; early to bed and arose early. Followed ordinary diet and had a calm mind. Mental faculties clear to the end.

13. A. S., farmer, 90 years old; worked hard and followed the usual diet, such as tea, coffee, meat and potatoes. Early to bed and arose early; no fads; mental faculties clear to end.

14. Rev. J. M., retired minister; died at 95; in full possession of all mental faculties; lived simply and on ordinary diet. Chewed food well and had a calm mind; early to bed and arose early. No fads of any kind.

15. R. T. A., minister; 103 years old; calm mind; no fads; followed the usual diet, smoked and took coffee all of his life.

16. D. P., 100 years old; had no fads of any kind; has lived plainly all of his life and had good health; full use of mental faculties.

17. Mrs. W., 100 years old; never had any fads; lives on ordinary diet and has good health.

18. W. N., 95 years old; never had any fads.

19. J. P., died at age of 86; never had any fads. Went to bed early and arose early. Lived plainly; had a calm mind and full use of mental faculties to the end.

---

CONSIDERATE.—Magistrate (to prisoner): If you were there for no dishonest purpose, why were you in your stocking feet? Prisoner: I heard there was sickness in the family.—*Punch.*

